# Endoscopic versus Percutaneous Biliary Drainage in Palliation of Advanced Malignant Hilar Obstruction: A Meta-Analysis and Systematic Review

**DOI:** 10.1155/2016/4726078

**Published:** 2016-08-25

**Authors:** Harsha Moole, Sirish Dharmapuri, Abhiram Duvvuri, Sowmya Dharmapuri, Raghuveer Boddireddy, Vishnu Moole, Prathyusha Yedama, Naveen Bondalapati, Achuta Uppu, Charan Yerasi

**Affiliations:** ^1^Division of General Internal Medicine, University of Illinois College of Medicine at Peoria, Peoria, IL, USA; ^2^Department of Internal Medicine, Wilkes-Barre Veterans Affairs Medical Center, Scranton, PA, USA; ^3^Department of Gastroenterology and Hepatology, Kansas City Veteran Affairs Medical Center, Kansas City, MO, USA; ^4^Division of General Internal Medicine, NTR University of Health Sciences, Andhra Pradesh, India; ^5^Division of Medicine, Barnes Jewish Christian Medical Group, Christian Hospital, St. Louis, MO, USA; ^6^Department of Medicine, Bronx Lebanon Hospital Center, Bronx, NY, USA; ^7^Department of Medicine, Albert Einstein College of Medicine, Bronx, NY, USA; ^8^Department of Medicine, MedStar Georgetown University Hospital and MedStar Washington Hospital Center, Washington, DC, USA

## Abstract

*Background*. Palliation in advanced unresectable hilar malignancies can be achieved by endoscopic (EBD) or percutaneous transhepatic biliary drainage (PTBD). It is unclear if one approach is superior to the other in this group of patients.* Aims*. Compare clinical outcomes of EBD versus PTBD.* Methods*.* (i) Study Selection Criterion*. Studies using PTBD and EBD for palliation of advanced unresectable hilar malignancies.* (ii) Data Collection and Extraction*. Articles were searched in Medline, PubMed, and Ovid journals.* (iii) Statistical Method*. Fixed and random effects models were used to calculate the pooled proportions.* Results*. Initial search identified 786 reference articles, in which 62 articles were selected and reviewed. Data was extracted from nine studies (*N* = 546) that met the inclusion criterion. The pooled odds ratio for successful biliary drainage in PTBD versus EBD was 2.53 (95% CI = 1.57 to 4.08). Odds ratio for overall adverse effects in PTBD versus EBD groups was 0.81 (95% CI = 0.52 to 1.26). Odds ratio for 30-day mortality rate in PTBD group versus EBD group was 0.84 (95% CI = 0.37 to 1.91).* Conclusions*. In patients with advanced unresectable hilar malignancies, palliation with PTBD seems to be superior to EBD. PTBD is comparable to EBD in regard to overall adverse effects and 30-day mortality.

## 1. Introduction

Malignant hilar strictures are primarily caused by hilar cholangiocarcinoma (HCCA); other differentials include local extension of gall bladder cancer, hepatocellular carcinoma, and metastasis from a distant primary site [1–3]. Geographically, HCCA is more prevalent in Asian countries, probably related to liver fluke infestation in these countries [4]. Majority of the patients (70–80%) with a hilar malignancy present late in the disease process, when curative surgical resection is no longer an option due to the extent of the disease [5]. Palliation is the goal in these patients.

Biliary obstruction alters the normal physiology and could affect multiple organ systems that include but are not limited to cardiac, renal, hematologic, and hepatic dysfunction [6–9]. Hyperbilirubinemia is a potential risk factor that might be associated with poor surgical outcomes and increases mortality [10–14]. Evidence suggests that biliary drainage may improve immune function and nutritional status and reduce risk of infection [15–17]. Biliary stenting is therefore a widely accepted method of palliation especially in patients with persistent pruritus, cholangitis, elevated bilirubin, and abdominal pain.

Biliary drainage can be achieved by internal or external approach. Internal biliary drainage is achieved by endoscopic retrograde placement of a biliary stent and endoscopic sphincterotomy. External biliary drainage is performed via fluoroguided percutaneous transhepatic approach that may later be internalized. Biliary drainage can also be achieved through surgical bypass; however, this is a less preferred approach due to invasiveness of the procedure.

In patients with Bismuth type I and II HCCA, it is a popular opinion that endoscopic biliary drainage (EBD) is preferred over percutaneous transhepatic biliary drainage (PTBD) as it is quick and comparatively less invasive. However, in patients with advanced unresectable hilar malignancies (including Bismuth types III and IV), it is unclear if one approach is superior to the other. Single centered studies comparing both these approaches have shown mixed results [18–26]. In this meta-analysis we aim to compare PTBD and EBD in patients with advanced unresectable hilar malignancies, to evaluate if one approach is superior to the other. Primary outcome was successful biliary drainage; secondary outcomes were overall adverse effects, cholangitis, pancreatitis, postpapillotomy bleeding, and 30-day mortality rate in both the groups. A subgroup analysis was performed on patients with advanced unresectable hilar cholangiocarcinoma, comparing the same outcomes.

## 2. Methods

### 2.1. Study Selection Criteria

#### 2.1.1. Inclusion Criteria

Studies using PTBD and/or EBD for palliation of advanced unresectable hilar malignancies and studies including patients with Bismuth type III and IV hilar cancers were included in this analysis.

#### 2.1.2. Exclusion Criteria

Studies including Bismuth type I and II HCCA were excluded. Studies including patients that underwent any surgical resection for their hilar malignancies were excluded from this analysis.

### 2.2. Data Collection and Extraction

Articles were searched in Medline, PubMed, and Ovid journals, EMBASE, Cumulative Index for Nursing & Allied Health Literature, ACP journal club, DARE, International Pharmaceutical Abstracts, old Medline, Medline nonindexed citations, Ovid Healthstar, and Cochrane Central Register of Controlled Trials (CENTRAL). The search was performed for the year 1966 to March 2016. Abstracts were manually searched in the major gastroenterology journals for the past 3 years. Study authors for the abstracts included in this analysis were contacted when the required data for the outcome measures could not be determined from the publications. The search terms used were endoscopic biliary drainage, percutaneous transhepatic biliary drainage, hilar malignancy, hilar cholangiocarcinoma, palliation, advanced hilar malignancies, and unresectable hilar malignancies. Two authors (HM and SP) independently searched and extracted the data into an abstraction form. Any differences were resolved by mutual agreement. The agreement between reviewers for the collected data was quantified using Cohen's *κ* [27].

### 2.3. Quality of Studies

Clinical trials designed with control and treatment arms can be assessed for quality of the study. A number of criteria have been used to assess this quality of a study (e.g., randomization, selection bias of the arms in the study, concealment of allocation, and blinding of outcome) [28, 29]. The study design for the present meta-analysis and systematic review was written in accordance with Meta-Analysis of Observational Studies in Epidemiology (MOOSE) study group. The present analysis conformed to the guidelines of PRISMA (Preferred Reporting Items for Systematic Reviews and Meta-Analyses) statement. PRISMA checklist is in Supplementary Material available online at http://dx.doi.org/10.1155/2016/4726078.

### 2.4. Statistical Methods

This meta-analysis was performed by calculating pooled proportions. First the individual study proportion was transformed into a quantity using Freeman-Tukey variant of the arcsine square root transformed proportion. The pooled proportion is calculated using inverse arcsine variance weights for the fixed effects model and DerSimonian-Laird weights for the random effects model [30, 31]. Forrest plots were drawn to show the point estimates in each study in relation to the summary pooled estimate. The width of the point estimates in the Forrest plots indicates the assigned weight to that study. The heterogeneity among studies was tested using *I* ^2^ and Cochran's *Q* test based upon inverse variance weights [32]. If *p* value is >0.10, it rejects the null hypothesis that the studies are heterogeneous. The effect of publication and selection bias on the summary estimates was tested by both Harbord-Egger bias indicator [33] and Begg-Mazumdar bias indicator [34]. Also, funnel plots were constructed to evaluate potential publication bias [35, 36]. Microsoft Excel 2013 software was used to perform statistics for this meta-analysis.

### 2.5. Outcome Measures

The definitions used in this analysis are the standard definitions used in almost all the studies included in this analysis. Successful biliary drainage was defined as a decrease in serum bilirubin levels to less than 50% of the pretreatment value or serum bilirubin levels < 2 mg/dL within two weeks after the drainage procedure [37]. Cholangitis was considered present when patient had fever, abdominal pain, and worsening biochemical parameters within 7 days of drainage [38]. Pancreatitis was diagnosed when serum lipase or amylase is three times above normal limits along with presence of abdominal pain for more than 24 hrs after procedure [38]. Significant bleeding was defined as a drop in hemoglobin level of >2 mg/dL or requirement for blood transfusion of more than 2 units or for a hemostatic procedure after a drainage procedure. Criteria for nonresectability of HCCA were taken from Aljiffry et al. [5].

## 3. Results

**Figure 1:**
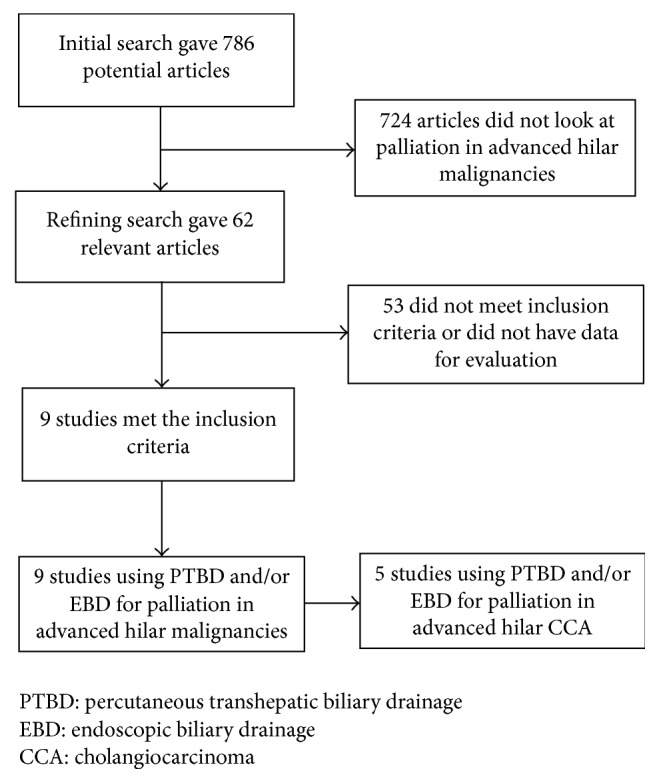
Flow diagram: search results.

Initial search identified 786 reference articles, in which 62 articles were selected and reviewed. Data was extracted from nine studies (*N* = 546) [18–26] using PTBD and/or EBD for palliation in advanced hilar malignancies, which met the inclusion criterion. All the studies are published as full-text articles.  Figure 1 shows the flow diagram of search results. All the pooled estimates given are estimates calculated using fixed effect model. Fixed effect model was preferred to random effects model for better accuracy based on the nature of individual study characteristics and heterogeneity. *p* value was >0.10, hence rejecting the null hypothesis that the studies are heterogeneous. Of the nine studies included in this meta-analysis, two are randomized controlled trials [21, 23] and seven are retrospective studies [18–20, 22, 24–26].

The total number of patients included in this meta-analysis is 546, with a predominant male population (*N* = 322). Five of the nine studies exclusively included patients with advanced HCCA [18, 20, 22, 24, 25]. A subgroup analysis was performed on these five studies (*N* = 357). There was one study each that exclusively included patients with hilar obstruction from hepatocellular carcinoma [26] and gall bladder cancer [23], respectively. Two studies [19, 21] included a mix of patients with hilar obstruction from various advanced malignancies (HCCA, hepatocellular carcinoma, and gall bladder cancer).  Table 1 shows the baseline characteristics of the studies. This article looked at various outcomes including successful biliary drainage, complications, and mortality. However, most of the studies did not have information on all the variables studied in this meta-analysis. Studies with pertinent information regarding a particular variable were included in calculating the pooled effect of that particular variable.

### 3.1. Successful Biliary Drainage

**Figure 2:**
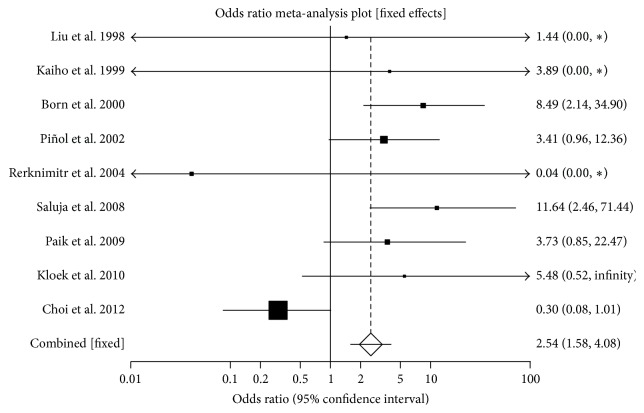
Forrest plot showing odds ratio of individual study proportion and pooled estimate comparing successful biliary drainage in PTBD versus EBD (fixed effects). ∗ refers to studies with single wing.

**Figure 3:**
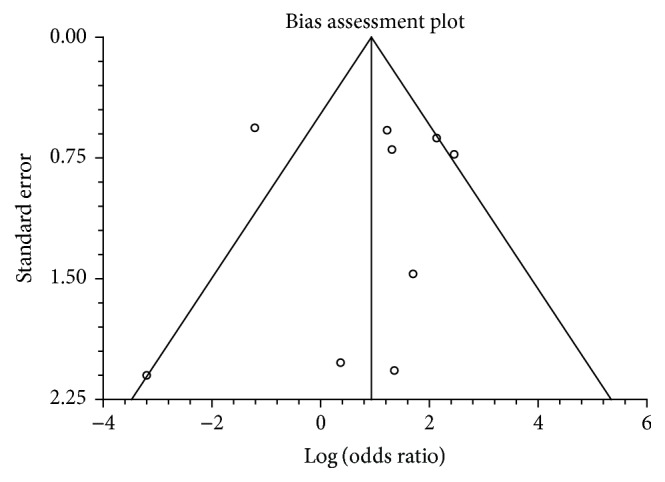
Funnel plot to evaluate publication bias (for successful biliary drainage in PTBD versus EBD).

The pooled odds ratio for successful biliary drainage in PTBD group compared to EBD group in patients with advanced hilar malignancy was 2.53 (95% CI = 1.57 to 4.08). Heterogeneity of studies was measured using *I* ^2^ (inconsistency) = 70.8% (95% CI = 29.1% to 83.6%). Egger: bias = −0.25 (95% CI = −3.83 to 3.33).  Figure 2 is a Forrest plot showing odds ratio of individual study proportion and pooled estimate comparing successful biliary drainage in PTBD versus EBD.  Figure 3 is a funnel plot for the successful drainage to evaluate publication bias.

### 3.2. Morbidity and Mortality

In patients with advanced hilar malignancies the odds ratio for overall adverse effects, cholangitis, pancreatitis, and postpapillotomy bleeding in PTBD versus EBD groups was 0.81 (95% CI = 0.52 to 1.26), 0.60 (95% CI = 0.36 to 0.99), 0.75 (95% CI = 0.30 to 1.84), and 5.39 (95% CI = 1.38 to 21.15), respectively. Odds ratio for 30-day mortality rate in patients of PTBD group versus EBD group was 0.84 (95% CI = 0.37 to 1.91).

### 3.3. Subgroup Analysis for Advanced HCCA

**Figure 4:**
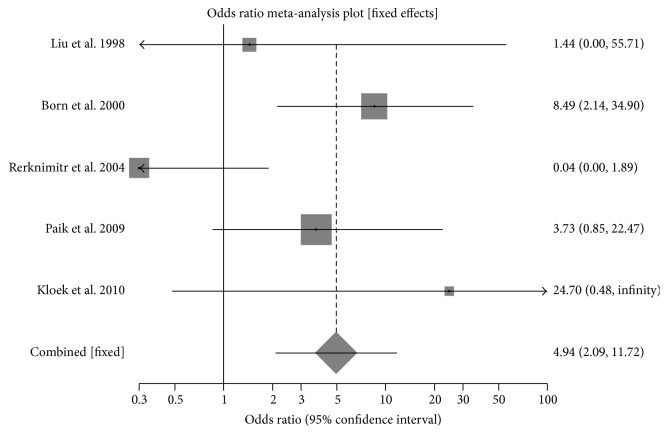
Forrest plot showing odds ratio of individual study proportion and pooled estimate comparing successful biliary drainage in PTBD versus EBD, advanced HCCA subgroup (fixed effects).

The pooled odds ratio for successful biliary drainage in PTBD group compared to EBD group was 4.94 (95% CI = 2.09 to 11.72). Odds ratio for overall adverse effects, cholangitis, pancreatitis, and postpapillotomy bleeding in PTBD versus EBD groups was 0.91 (95% CI = 0.49 to 1.68), 0.51 (95% CI = 0.24 to 1.08), 1.36 (95% CI = 0.42 to 4.39), and 9.41 (95% CI = 1.56 to 56.59), respectively. Odds ratio for 30-day mortality rate in PTBD group versus EBD group was 1.82 (95% CI = 0.33 to 10.03). Heterogeneity of studies was measured using *I* ^2^ (inconsistency) = 41% (95% CI = 0% to 77.1%). Egger: bias = −1.13 (95% CI = −4.61 to 2.36).  Figure 4 is a Forrest plot for this subgroup showing odds ratio of individual study proportion and pooled estimate comparing successful biliary drainage in PTBD versus EBD.

## 4. Discussion

There are advantages and disadvantages for both endoscopic retrograde cholangiopancreatography (ERCP) guided endoscopic biliary drainage and percutaneous transhepatic biliary drainage. PTBD facilitates precise lobar selection along with reducing the risk of exposing the biliary tree to duodenal contents. This would conceptually increase the success of biliary drainage and reduce the risk of cholangitis [39]. Performing PTBD requires minimal sedation, hence feasible in unstable patients who cannot tolerate anesthesia [40]. PTBD is associated with pain and discomfort at the skin puncture site. Sometimes, PTBD should be followed up by internalization of stent that may be associated with increased infection and bleeding complications [40, 41]. EBD is a less invasive approach with superior outcomes in patients with Bismuth type I and II HCCA [22, 41]. However, its role in advanced malignant hilar obstruction is controversial. Biliary hilar obstruction stenting is considered to be a complex and difficult endoscopic procedure. American Society of Gastrointestinal Endoscopy (ASGE) grades ERCPs as levels 1 through 4, level 4 being the most complex and level 1 being the simplest [42]. Hilar biliary stenting is recognized as level 3 and therefore associated with increased complication rates and lower success rate [43]. Hence, hilar stenting should only be performed by skilled and experienced advanced endoscopists.

Self-expandable metal stents (SEMS) are preferred over plastic stents (PS) due to longer stent patency, fewer reinterventions, shorter hospital stay, and cost-effectiveness. PS may be used when life expectancy of the patient is less than 4–6 months [40]. Vienne et al. suggested that drainage of >50% of the liver resulted in better outcomes [44]. Therefore, it may be reasonable to use more than one stent to achieve this.

Choi et al. [26] included patients with unresectable hepatocellular carcinoma, indicating that EBD is preferable to PTBD due to longer duration of drainage patency. Kloek et al. [25], Paik et al. [24], and Piñol et al. [21] concluded that PTBD is superior to EBD in patients with advanced HCCA. Saluja et al. [23] concluded that PTBD is preferable to EBD in palliation of hilar obstruction caused by gall bladder cancer.

This meta-analysis showed that PTBD group has significantly higher drainage success compared to EBD group. Patients that underwent PTBD had relatively less cholangitis episodes; however, there was no significant difference in pancreatitis and overall complications in both groups. 30-day mortality period was comparable in both the groups; this could probably be due to the underlying nature of the disease itself. PTBD group however had increased postprocedure bleeding complications. This might be due to the second step of the PTBD approach in some patients that involves internalization of the stent.

Based on the results of this analysis, in patients with advanced unresectable hilar malignancies, PTBD appears to be superior to EBD with higher rates of successful drainage and less frequent cholangitis. However, patients should be watchfully monitored for post-PTBD bleeding complications, especially after internalization of the stent. This being said, the final decision on the choice of palliation depends on multiple factors: availability of an experienced advanced endoscopist, comorbidities of the patient, patient preference, stage, and Bismuth type of the hilar malignancy, life expectancy of the patient, and patient's ability to tolerate anesthesia. The presence of an external percutaneous catheter is a very significant issue for most patients and an important factor in their decision-making during informed consent.

There are a few limitations of this study. Articles included in this analysis have used different stents: plastic stents and self-expandable metal stents. Plastic stents are usually associated with increased number of complications, reintervention rate, and short patency. Only PS were used in four studies [18, 23, 25, 26], only SEMS were used in two studies [19, 24], and a mix of both SEMS and PS was used in three studies [20–22]. Based on the availability of skillful advanced endoscopists, operator variability might affect the EBD outcomes. Of the nine studies included in the meta-analysis, only two were RCTs [21, 23] and the rest were retrospective studies. We were not able to evaluate quality of life, cost benefit analysis, and stent patency periods in both groups due to the sporadic data available from individual studies. With just 9 studies, funnel plot asymmetry may not be an accurate assessment for the presence of bias.

Strengths of this meta-analysis include the high quality methodology of statistical analysis, high quality methodology used in individual studies, total number of patients included in this analysis (*N* = 546), and the fact that we were able to perform a subgroup analysis on patients with advanced HCCA.

Studies with statistically significant positive results tend to be published and cited. Additionally, smaller studies may show larger treatment effects compared to larger studies. This publication and selection bias may affect the summary estimates. The bias can be estimated using Egger bias indicators and the construction of funnel plots, whose shape can be affected by bias. In the present meta-analysis and systematic review, bias calculations of both Harbord et al. [33] and Begg and Mazumdar [34] bias indicators showed no statistically significant bias. Furthermore, analysis using funnel plots showed no significant publication bias among the studies included in the present analysis.

Other promising prospects for palliation include endoscopic ultrasound (EUS) guided biliary drainage [45–48], photodynamic therapy [49–51], radiofrequency ablation [52, 53], and palliative chemotherapy/radiation therapy [54–58]. There are a few single centered and multicentered studies that showed better outcomes with these approaches, suggesting that they could be considered in advanced tumors (Bismuth types III and IV) in addition to regular stenting. There is ongoing research in these fields. Further properly powered RCTs looking at these topics might provide more efficient approaches for palliation.

## 5. Conclusions

In patients with advanced unresectable hilar malignancies, palliation with PTBD seems to be superior to EBD. PTBD was associated with higher rates of successful biliary drainage and lower rates of cholangitis. EBD has lower bleeding complications compared to PTBD. PTBD was comparable to EBD in regard to overall adverse effects and 30-day mortality rate.

## Supplementary Material

Supplementary material is a PRISMA checklist

## Figures and Tables

**Table 1:** Basic characteristics of the included studies.

Number	Name	Type of study	Type of drainage	Number of patients	Number of patients in PTBD group	Number of patients in EBD group	M/F	Age in years, median	Location of stricture	Type of cancer
1	Liu et al. 1998 [18]	Retrospective	Endoscopic	49	0	49	33/16	68	Hilum	CCA
2	Kaiho et al. 1999 [19]	Retrospective	Percutaneous	21	21	0	9/12	67	Hilum	All cancers
3	Born et al. 2000 [20]	Retrospective	Both	59	39	20	30/29	71	Hilum	CCA
4	Piñol et al. 2002 [21]	RCT	Both	54	28	26	23/31	73	Mixed	All cancers
5	Rerknimitr et al. 2004 [22]	Retrospective	Endoscopic	63	0	63	35/28	65	Hilum	CCA
6	Saluja et al. 2008 [23]	RCT	Both	54	27	27	18/36	51	Hilum	Gall bladder cancer
7	Paik et al. 2009 [24]	Retrospective	Both	85	41	44	58/27	66	Hilum	CCA
8	Kloek et al. 2010 [25]	Retrospective	Both	101	11	90	70/31	61	Hilum	CCA
9	Choi et al. 2012 [26]	Retrospective	Both	60	31	29	46/14	59	Hilum	HCC

CCA: cholangiocarcinoma.

HCC: hepatocellular carcinoma.

M/F: male/female.

RCT: randomized controlled trial.

EBD: endoscopic biliary drainage.

PTBD: percutaneous transhepatic biliary drainage.
